# INTEGRATING PRINCIPLES OF VALUE-BASED CARE THROUGH COMMUNITY-BASED ORGANIZATION PARTNERSHIPS

**DOI:** 10.1093/geroni/igz038.1361

**Published:** 2019-11-08

**Authors:** Marla Berg-Weger, Catherine Taylor

**Affiliations:** 1 Saint Louis University, St. Louis, Missouri, United States; 2 The George and Anne Ryan Institute for Neuroscience The Rhode Island Geriatric Education Center The University of Rhode Island, Providence, Rhode Island, United States

## Abstract

To develop programming consistent with value-based care principles, two GWEPs share strategies and policy implications when partnering with community-based organizations (CBO). Because the aims of value-based care – better patient outcomes and population health, reduced health care costs – cannot be achieved by changing medical practice alone, RI Geriatric Education Center (RIGEC) partners with multiple CBOs to deliver evidence-based patient education programming to empower patients/caregivers to take charge of their health. RIGEC will work directly with physician practices to bring education directly to their high-risk patient census. Saint Louis University CBO partners integrated geriatric assessment into routine care to identify geriatric syndromes. Introduction of protocols for Medicare Annual Wellness Visits, Cognitive Stimulation Therapy and exercise/strengthening enables CBOs to offer interventions. Value-based care is achieved by reducing costs as geriatric syndromes are identified earlier, enabling diagnosis, treatment, and management; improving patient outcomes and satisfaction; and strengthening provider competence in geriatric care.

